# Establishment of mouse neuron and microglial cell co-cultured models and its action mechanism

**DOI:** 10.18632/oncotarget.17898

**Published:** 2017-05-16

**Authors:** Bo Zhang, Yunfeng Yang, Jun Tang, Yihao Tao, Bing Jiang, Zhi Chen, Hua Feng, Liming Yang, Gang Zhu

**Affiliations:** ^1^ Department of Neurosurgery, Southwest Hospital,Third Military Medical University, Chongqing, China

**Keywords:** neuron, microglial cell, OGD, tOGD

## Abstract

**Objective:**

The objective of this study is to establish a co-culture model of mouse neurons and microglial cells, and to analyze the mechanism of action of oxygen glucose deprivation (OGD) and transient oxygen glucose deprivation (tOGD) preconditioning cell models.

**Results:**

Mouse primary neurons and BV2 microglial cells were successfully cultured, and the OGD and tOGD models were also established. In the co-culture of mouse primary neurons and microglial cells, the cell number of tOGD mouse neurons and microglial cells was larger than the OGD cell number, observed by a microscope. CCK-8 assay result showed that at 1h after treatment, the OD value in the control group is lower compared to all the other three groups (*P* < 0.05). The treatment group exhibited the highest OD value among the four groups. The results observed at 5h were consistent with the results at 1 h. Flow cytometry results showed that at 1h after treatment the apoptosis percentages is higher in the control group compared to other three groups (*P* < 0.05).

**Materials and Methods:**

Mouse brain tissues were collected and primary neurons cells were cultured. In the meantime mouse BV2 microglia cells were cultured. Two types of cells were co-cultured, and OGD and tOGD cell models were established. There were four groups in the experiment: control group (OGD), treatment group (tOGD+OGD), placebo group (tOGD+OGD+saline) and minocycline intervention group (tOGD+OGD+minocycline). CCK-8 kit was used to detect cell viability and flow cytometry was used to detect apoptosis.

**Conclusions:**

In this study, mouse primary neurons and microglial cells were co-cultured. The OGD and tOGD models were established successfully. tOGD was able to effectively protect neurons and microglial cells from damage, and inhibit the apoptosis caused by oxygen glucose deprivation.

## INTRODUCTION

Ischemic stroke, a common clinical disease also known as cerebral infarction, is most likely caused by various reasons and exhibits blockage of cerebral circulation supply, cerebral ischemia and hypoxia, leading to necrosis, as well as the subsequent neuronal functional damage [1]. Cerebral infarction is one of the main causes of death in clinical practice, with poor prognosis and causing a heavy burden of disease [2]. The current treatment options for cerebral infarction are limited. Ineffectiveness, high cost and significant individual differences in response are present in some of the therapies [3, 4]. Therefore, it has become a hot research topic to search an effective therapy for ischemic stroke.

Oxygen and glucose deprivation (OGD) is a model to simulate human brain infarction by depriving carbohydrates and oxygen from nutrient supply in the cells [5–8]. At present a number of studies have utilized OGD *in vitro* models to investigate an effective treatment method for ischemic stroke and have shown good results [9, 10]. Oxygen and glucose deprivation preconditioning refers to the tolerability of tissue to oxygen and glucose deprivation damage, acquired by once or multiple times of transient oxygen and glucose deprivation [11–13].

Microglia are resident macrophages in the brain, and are thought to be the major promoter and participant of the brain inflammatory response [14]. Its activation process includes cell proliferation, differentiation, phagocytosis and secretion of cytokines. Recent studies suggest that microglial function depends on the type of stimulation and the different activation states caused by the intensity and the corresponding different immune functional phenotypes, which can simultaneously exert neuroprotective and neurotoxic effects [14, 15]. In this study, mouse neurons and microglial cells were co-cultured, and OGD and tOGD models were established to investigate the protective effect and the mechanism of action of oxygen glucose deprivation preconditioning on neurons.

## RESULTS

### Co-culture of mouse neurons and BV2 microglial cells

**Figure 1:**
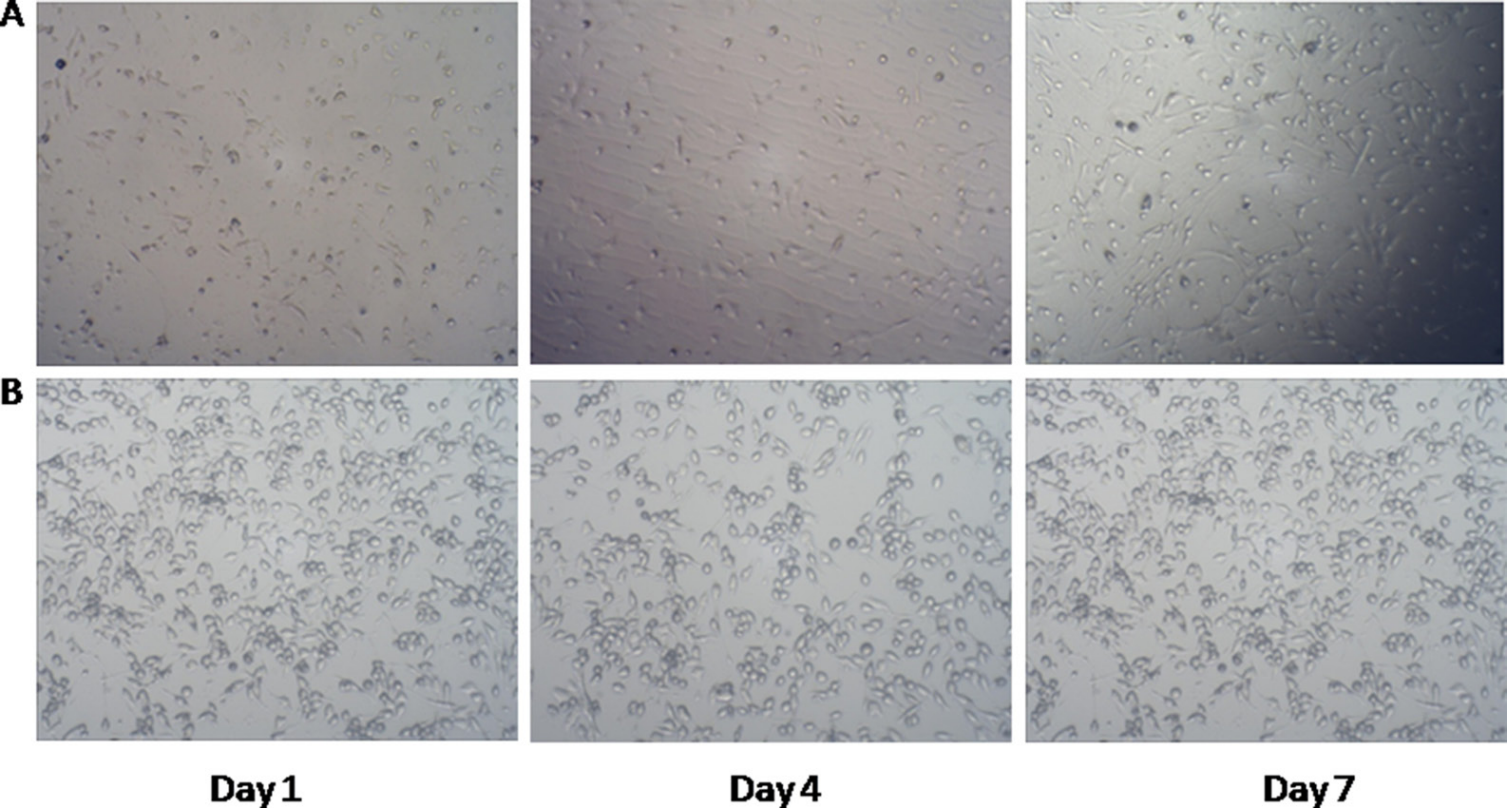
Mouse primary neuronal culture and BV2 microglial cell culture (**A**) Mouse Primary Neuronal Culture (100×); (**B**) Mouse BV2 Microglial Cell Culture (100×).

Mouse primary neurons and BV2 microglial cells were successfully cultured in the study. Cells grew in a good condition and were ready for subsequent experiments, as shown in Figure 1.

### Co-culture of two types of cells and OGD/tOGD models

**Figure 2:**
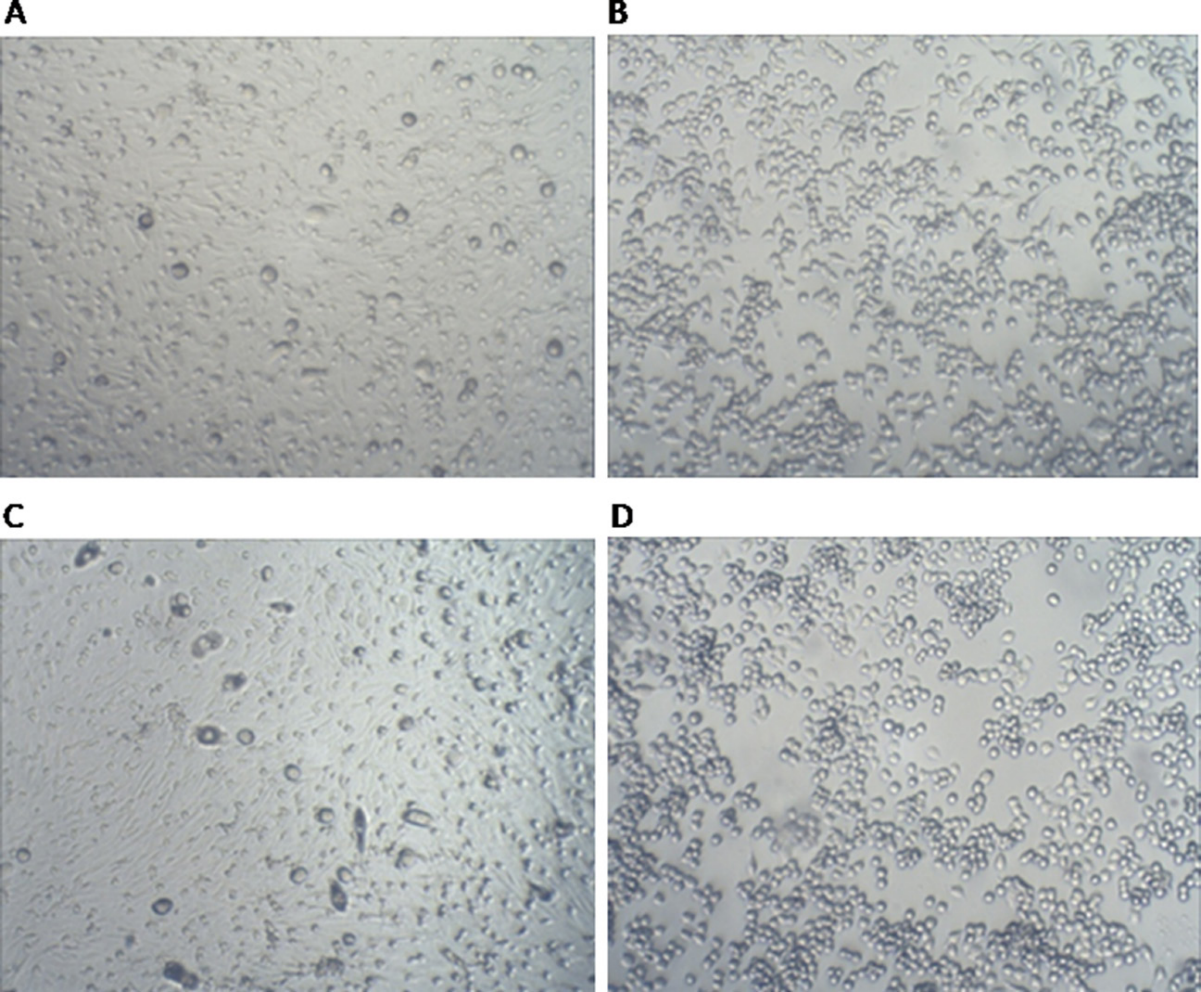
Cell co-culture and tOGD/OGD cell model (**A**) Neurons Cells with tOGD 1 h (100×); (**B**) BV2 Cells tOGD 1 h (100×); (**C**) Neurons cells with OGD 5 h (100×); (**D**) BV2 Cells with OGD 5 h (100×).

Mouse neurons and microglial cells were co-cultured and went through tOGD and OGD experiment. In the same sight of microscope, the number of tOGD mouse neurons and microglial cells was larger than the number of cells under OGD condition, as shown in Figure 2.

### Result of CCK-8 cell viability assay

**Figure 3:**
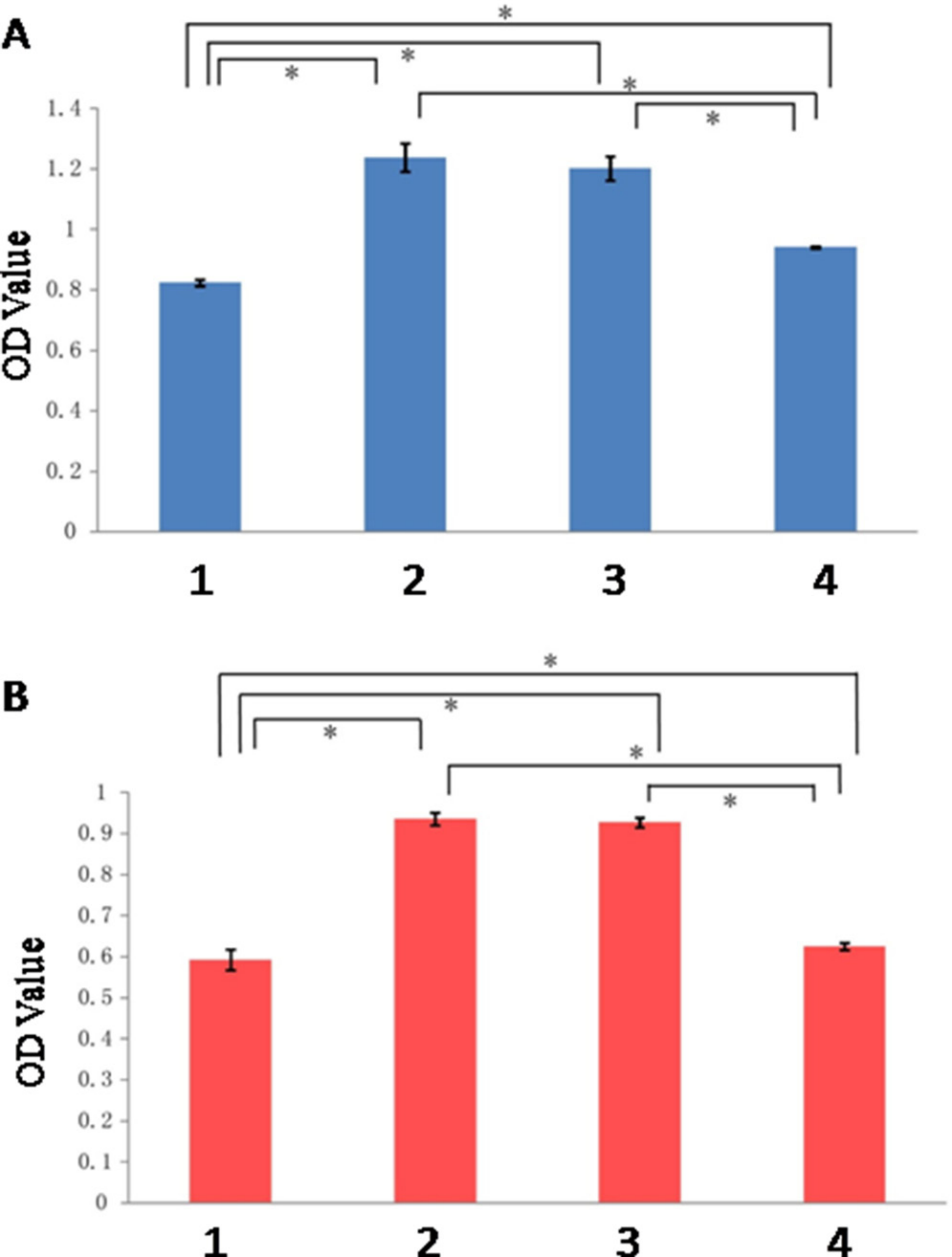
Result of 1 h and 5 h after OGD (**A**) 1 h; (**B**) 5 h. (1: Control Group; 2:Treatment Group; 3: Placebo Group; 4: Minocycline Intervention Group). *The difference between two groups was statistically significant (*P* < 0.05).

The result of CCK-8 assay showed that the difference of OD values between control group and the other three groups was statistically significant, with the OD values of all three groups higher compared to control group (*P* < 0.05). In the meantime, the difference between treatment group and minocycline intervention group, and that between placebo group and minocycline intervention group, were both statistically significant (*P* < 0.05). Among the four experimental groups, treatment group exhibited the highest OD value, as shown in Figure 3A. The result observed at 5 h was similar to that at 1 h, with the OD values of all three groups higher compared to control group (*P* < 0.05). The difference between treatment group and minocycline intervention group, and that between placebo group and minocycline intervention group, were both statistically significant (*P* < 0.05), as shown in Figure 3B.

### Result of apoptosis detection

**Figure 4:**
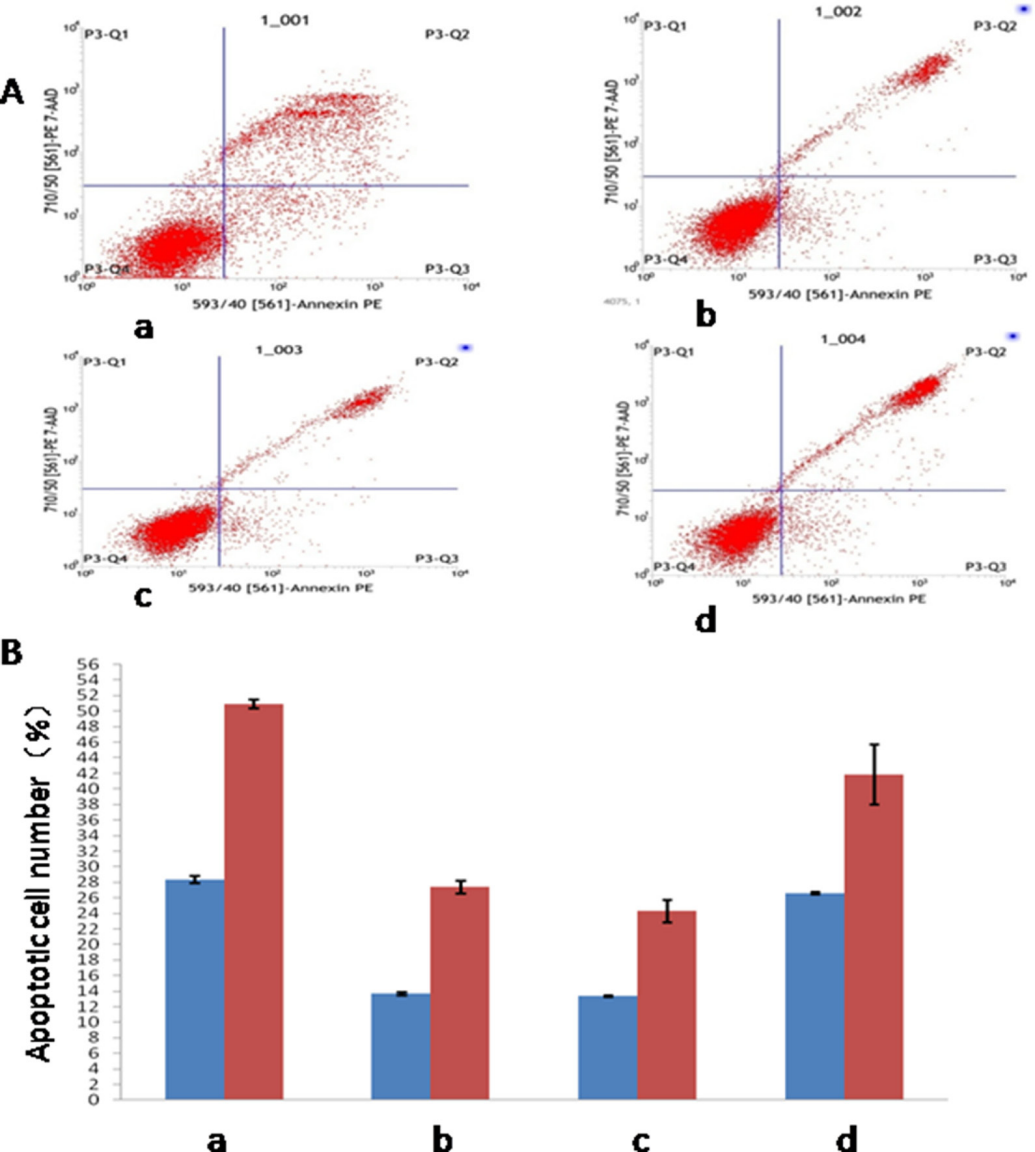
Detection of apoptosis in four experimental groups at different time points (**A**) Detection of Apoptosis by Flow Cytometry; (**B**) Apoptosis in different time point. (a: Control Group; b:Treatment Group; c: Placebo Group; d: Minocycline Intervention Group).

Flow cytometry was used to detect apoptosis of four experimental groups at 1 h and 5 h, as shown in Figure 4A. The results showed that at 1 h, the difference of apoptosis percentages between control group and the other three groups were statistically significant (*P* < 0.05), with the apoptosis percentages of all three groups lower compared to control group. In the meantime, the difference between treatment group and minocycline intervention group, and that between placebo group and minocycline intervention group, were both statistically significant (*P* < 0.05). The result observed at 5 h was similar to that at 1h, with the apoptosis percentage of all three other groups lower than that of control group (*P* < 0.05). The difference of apoptosis percentages between treatment group and minocycline intervention group, and that between placebo group and minocycline intervention group, were both statistically significant (*P* < 0.05), as shown in Figure 4B.

## DISCUSSION

In this study, we successfully co-cultured the mouse primary neurons and microglial cells. We also established the OGD and tOGD models. The results suggested that tOGD was able to effectively protect neurons and microglial cells from damage, and inhibit the apoptosis caused by oxygen glucose deprivation.

The research of OGD models has been reported previously and many mature methods have been established [14–16]. The preparation of OGD models includes two parts: oxygen deprivation and glucose deprivation. Oxygen deprivation is achieved by various methods to lower the oxygen to a minimum amount or undetectable level, while glucose deprivation is conducted by cell culture in glucose free media [17, 18]. In this study, cells were cultured in media without glucose, and oxygen was substituted with nitrogen to achieve an oxygen free condition. This is currently a mature method to prepare OGD models. It can be seen from our result that the OGD and tOGD models were successfully established in the mouse neuron and microglial cell co-culture model.

On the other hand, previous research on the mechanism of action of OGD models less involved apoptosis, while more focused on cell signal transduction and gene expression regulation [19–22]. In our study, we analyzed the neuronal damage resulted from OGD in terms of cellular viability and apoptosis, as well as the effect of tOGD on OGD. The results showed that tOGD effectively suppressed the effect of OGD on neurons and microglial cells. Most of neurons and microglial cells were able to grow and proliferate normally following the action of tOGD. In the meantime, preconditioning improved the cell survival rate, shown as cell proliferation rate elevation. tOGD also effectively reduced apoptosis caused by OGD. Therefore, from this perspective, we believed that tOGD might play an important role by inhibiting apoptosis led by OGD.

Two limitations existed in the present study: on the one hand, the mechanism of the effect of tOGD on the cell apoptosis was not be analyzed in depth. One the other hand, we did not performed experiment *in vivo*.

In conclusion, we found tOGD was able to effectively protect neurons and microglial cells from damage, and inhibit the apoptosis caused by oxygen glucose deprivation, based on the successful establishment of the OGD and tOGD models.

## MATERIALS AND METHODS

### Primary culture of mouse neurons

Forty SPF neonatal mice were purchased from the Experimental Animal Center of the Third Military Medical University. The healthy ICR neonatal mice were sterilized with 75% alcohol within 24 h after birth and decapitated in sterile condition. The scalp and skull were cut. Brain tissue was taken out and placed in a dish containing cold D-Hank's solution with pH7.2 and free of calcium and magnesium. Brain tissue was peeled in sterile condition. Cerebellum, hippocampus and medulla were removed. Cortex was isolated. Meninges and blood vessels were carefully peeled off. Tissue was cut into blocks of about 1 mm^3^ using iris scissors, digested in 0.125% trypsin for 20 min at 37°C and shaken two to three times. The supernatant was discarded and complete media was added to terminate digestion. Tissues were rinsed twice and pipetted 20 times slightly using the Pasteur pipette. The cell suspension was allowed to stand for 2 min. Cell suspension was collected in a new centrifuge tube and centrifuged at 1000 r/m at 4°C for 10 min. The supernatant was discarded. Cells were resuspended in complete media and filtered with a 200 mesh stainless steel filter. The filtrate was stained using trypan blue and cells were counted using a hemocytometer under a microscope. Cells were inoculated at an intensity of 8.0 × 10^4^ to 1.0 × 10^5^ ml with 400 μl/well into a 24-well plate or 100 μl/well into a 96-well plate pre-coated with poly-L-lysine. Cells were cultured for 4–6 h in an incubator at 37°C with 5% CO^2^. Media was changed with serum-free media. At day 3 of *in vitro* culture, Ara-C working solution was added to inhibit over-proliferation of non-neuronal cells, and aspirated after 24 h treatment. Thereafter media was changed by half every three days. Cells collected from day 7–21 of *in vitro* culture were used for the experiment.

### Cell culture of mouse microglial cells

Mouse BV2 microglial cell line was purchased from the cell bank of Chinese Academy of Science, Shanghai. Cryovials were taken out of the **−**80°C freezer or liquid nitrogen tank, quickly placed into 37°C water bath, and shaken mildly to facilitate the contents thawing. Cell suspension was aspirated from cryovials and added into a centrifuge tube with 5 ml cell culture media containing 10% fetal bovine serum (FBS). Cells were centrifuged at 800 to 1000 rpm for 5 min and the supernatant was discarded. Cells was added with 1ml culture media and pipetted to make suspension. Cells were diluted in cell media containing 20% FBS, inoculated into a cell culture flask, placed in an incubator at 37°C with saturated humidity and 5% CO_2_. Culture media was changed after the first 24 h and the subsequent change was determined based on cell growth condition. Adherent cells were observed under a microscope. With 70% to 80% confluence, old media was removed from flask and cells were rinsed with PBS for two to three times. 1Trypson was added to digest cells following a ratio of 1–3 ml tryspin for a 50 ml flask. When cells became shrank, round and detached in the microscope, the flask was shaken slightly to detach all cells. 2–3 ml complete media was added and pipetted. Cells were collected in a centrifuge tube. The supernatant was removed and culture media was added. Cells were pipetted slightly into cell suspension and inoculated into a sterile culture flask with media following 1:2 or 1:3 ratio. Cell culture was continued or used for further experiments.

### Co-culture of two types of cells model establishment of OGD and tOGD

BV2 cells were cultured in DMEM high glucose media containing 10% FBS, at 37°C in an incubator with CO_2_ gas. Cells were sub-cultured every 2–3 days. Cells from passage 3 to 6 were collected for subsequent experiments. Neurons were seeded in different dishes and cells from day 7 to 21 of *in vitro* culture were used for experiment. Co-culture and transwell culture were conducted depending on the purpose of the experiments. Neurons and BV2 were co-cultured following a 1:2 ratio. 96-well plates were seeded with neurons 3 × 10^4^/well and BV2 cells 1.5 × 104/well, placed in an incubator at 37°C with saturated humidity and 5% CO_2_.

OGD and tOGD models: after cell seeding, the incubator was filled with nitrogen to substitute air and the subsequent experiments were not conducted until a stable condition of 95% nitrogen and 5% CO_2_. Cells were placed in the above condition. The original culture media was changed into DEME media without glucose (Gibco). Cells growing in the original culture condition were used as positive control. Cells were cultured in tOGD condition for 1 h, and in OGD condition for 5 h. Normal culture media was used for 24 h in the normal condition between the two cultures.

### Detection of cell viability by CCK-8

Cell counting kit (CCK-8 kit) was used to detect cell viability and cells were cultured in the same condition as above. There were four groups in the experiment: control group (OGD), treatment group (tOGD+OGD), placebo group (tOGD+OGD+saline) and minocycline intervention group (tOGD+OGD+minocycline). Cells from different treatment groups were counted, adjusted the concentration to 1 × 10^5^ ml, and seeded in a 96-well plate with 100 ul/well. Cells were seeded in triplicate for each treatment group. The 96-well plate was placed in the incubator (37°C and 5% CO2) and cells were cultured till the appropriate time. 10 μl CCK-8 solution was added in each well and the cell culture plate was incubated for 1–4 h. The absorbance at 450 nm was detected using a plate reader. Blank wells (culture media and CCK) and control wells (untreated cells, culture media and CCK) were also detected.

### Detection of apoptosis

Cell culture and experiment groups were set up as above. Cells were cultured in a 6-well plate till 60% to 70% confluence. Based on the needs of experiment, cell culture was continued. Cells were aspirated and placed in a centrifuge tube, centrifuged at 2000 rpm for 5min, rinsed with PBS twice and centrifuged again at 2000 rpm for 5 min. 1 × 10^5^ cells were collected. 5 μl 7-AAC staining solution was added in the 50μl binding buffer and mixed. The above 7-ADD staining solution was added in the collected cells and placed in the dark at room temperature for 5–15 min reaction. 450 μl binding buffer was later added and mixed. 1 μl Annexin V-PE was added, mixed and placed in the dark at room temperature for 5–15 min. Fluorescent microscope observation or flow cytometry was conducted within 1 h. For flow cytometry, FL2 channel was used to detect the orange-red fluorescence of Annexin V-PE, with excitation wavelength of 488 nm and emission wavelength of 578 nm. FL3 channel was used to detect the red fluorescence of 7-AAD, with excitation wavelength of 546 nm and emission wavelength of 647 nm. Normal cells without apoptotic induction were used as control for fluorescence compensation to remove spectral overlap and determine the position of cross-over.

### Statistical analysis

SPSS17.0 software was used to conduct the statistical analysis. Qualitative variables were detected using chi-square test, while quantitative variables were detected by analysis of variance. Difference with *P* < 0.05 was considered statistically significant.
